# Multidisciplinary Team Care for Early‐Stage Lung Cancer in Low‐ and Middle‐Income Countries and Emerging Economies: Current Position and Call for Action

**DOI:** 10.1111/1759-7714.70372

**Published:** 2026-08-18

**Authors:** Luiz Henrique Araujo, Francisco J. Orlandi, Adli Azam, Lucia Viola, Yasser Elkerm, Jorge Alatorre‐Alexander, Sevin Başer Öncel, Gustavo A. Lyons, Xiao Li, Trung Lam Quoc, Anusheel Munshi, Francisco Gonzalez

**Affiliations:** ^1^ Department of Medical Oncology Instituto Nacional de Câncer Rio de Janeiro Brazil; ^2^ Lung Cancer Unit National Thorax Institute Santiago Chile; ^3^ Department of Cardiothoracic Surgery Gleneagles Hospital Johor Iskandar Puteri Malaysia; ^4^ Department of Cardiovascular and Thoracic Surgery, Faculty of Medicine Universiti Teknologi MARA (UiTM) Shah Alam Malaysia; ^5^ Center of Expertise in Thoracic Oncology Oncotórax, Fundación Neumológica Colombiana, Luis Carlos Sarmiento Angulo Cancer Treatment and Research Center Bogotá Colombia; ^6^ Department of Clinical Oncology Alexandria University Alexandria Egypt; ^7^ Medical Oncology Department Instituto Nacional de Enfermedades Respiratorias (INER) Mexico City Mexico; ^8^ Department of Pulmonary Medicine Pamukkale University Denizli Türkiye; ^9^ Thoracic Oncology Center, British Hospital Buenos Aires Argentina; ^10^ Department of Thoracic Surgery Peking University People's Hospital Beijing China; ^11^ Faculty of Medicine Nguyen Tat Thanh University Ho Chi Minh City Vietnam; ^12^ Chemotherapy Department Hospital of Medical University Ho Chi Minh City Vietnam; ^13^ Department of Radiation Oncology BLK MAX Hospital New Delhi India; ^14^ Medical Affairs, AstraZeneca Baar Switzerland

**Keywords:** early‐stage, low‐ and middle‐income countries, lung cancer, multidisciplinary team, recommendations

## Abstract

Lung cancer (LC) is a complex cancer marked by poor prognosis. Low‐ and middle‐income countries (LMICs) and emerging economies have witnessed a growing burden of LC in recent years due to low symptom awareness, rising tobacco use, air pollution, and pulmonary infections. Managing LC optimally requires a multidisciplinary team (MDT) approach to support accurate diagnosis, staging, and treatment. MDT presentation has been linked to timely diagnosis and staging, receipt of surgery, and survival benefits in early‐stage LC (ESLC). However, delivering high‐quality ESLC care through an MDT approach remains challenging in LMICs and emerging economies. Weak organizational structure, fragmentation of the financing and health system, oncology workforce deficits, and unawareness of the potential impact of MDTs hinder optimal care. Systematic identification, analysis, and reporting of barriers to ESLC care are critical for improving care quality and informing effective corrective interventions. This article aimed to explore barriers to MDT implementation that are unique to the care of patients with ESLC in LMICs and emerging economies and developed evidence‐informed recommendations for strengthening MDT care pathways. Key focus areas addressed by these recommendations include the composition of the MDT panel for ESLC management, standardizing MDT processes and guidelines, defining standardized datasets for data collection and internal MDT review, data sharing practices, and streamlining decision‐making processes. This guidance document also provides a rational framework for improved standardized MDT care in ESLC (RISE‐LC) to guide the implementation and optimization of MDT care in resource‐constrained settings, aiming to improve timely diagnosis, treatment coordination, and patient outcomes.

## Graphical abstract

Multidisciplinary team (MDT) care improves timeliness, surgical treatment, and survival in early‐stage lung cancer. However, existing international guidelines are not tailored to low‐ and middle‐income countries. This article identifies region‐specific challenges and provides recommendations for standardized MDT care in resource‐constrained settings.

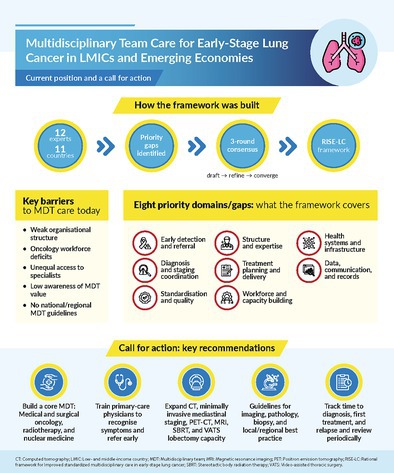

## 1 Introduction

**FIGURE 1:**
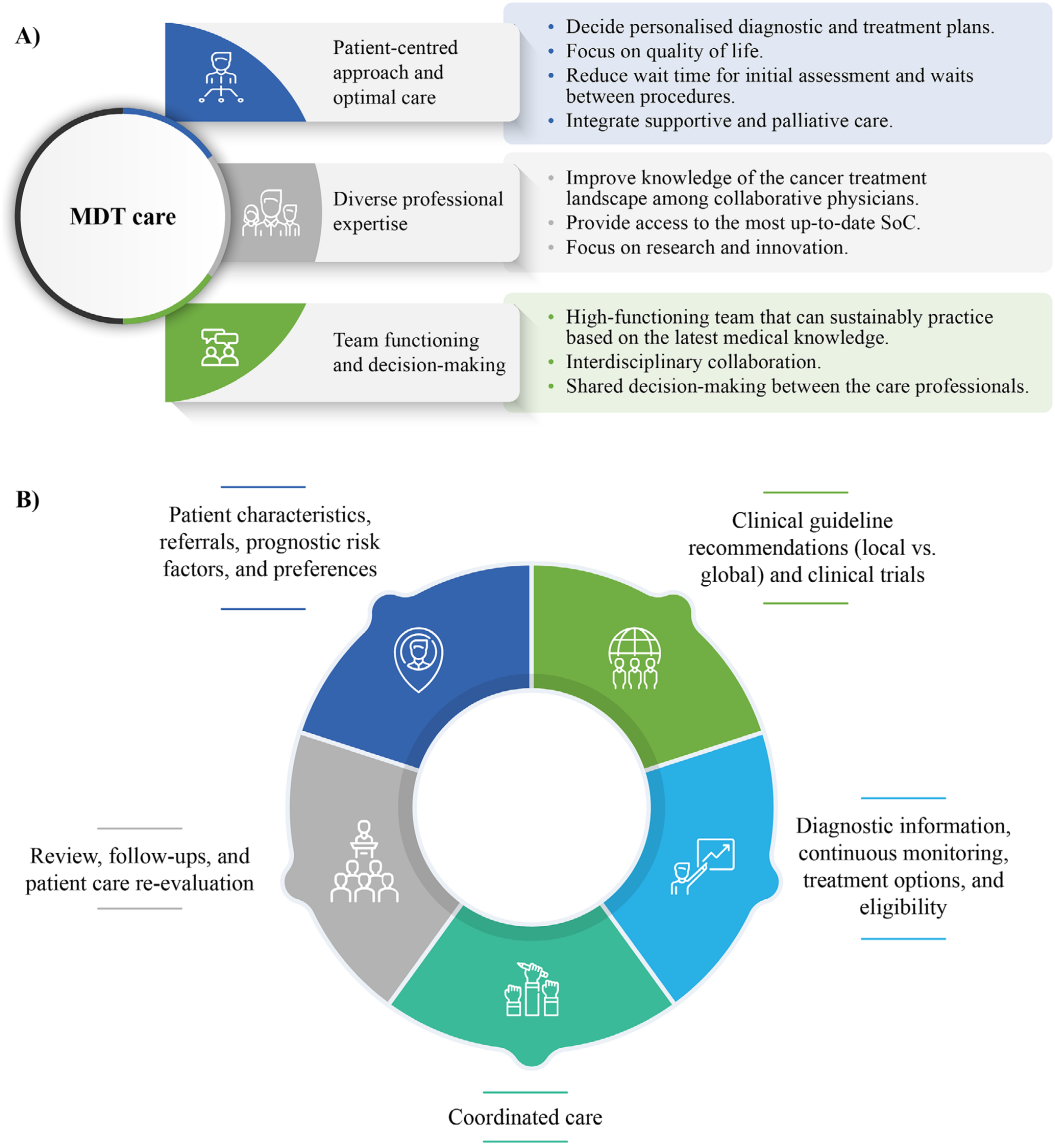
Key elements of the MDT approach to LC care (A) and considerations of the MDT for establishing optimal care pathways (B). LC, lung cancer; MDT, multidisciplinary team; SoC, standard of care. *Source:* Lubuzo et al. [13], Lubuzo et al. [14], Denton et al. [16], Liu et al. [17], and Hardavella et al. [18].

Lung cancer (LC) is the most frequently diagnosed cancer and the leading cause of cancer‐related deaths globally [1]. As per the Global Cancer Observatory 2022 estimates, LC was responsible for nearly one in eight (12.4%) cancers diagnosed and one in five (18.7%) cancer deaths [2]. Non‐small cell LC (NSCLC) accounts for 80%–85% of LC cases [1]. LC is a complex cancer characterized by a lack of early noticeable symptoms, leading to diagnostic/treatment delays and poor outcomes [1, 2, 3, 4, 5, 6]. The survival rate of early‐stage LC (ESLC) varies by stage, with a 5‐year survival rate (clinical staging; American Joint Committee on Cancer, 8th edition) of 92% for stage IA1 and < 60% for stage II [7]. The survival rate is 36% for stage IIIA NSCLC [8]. LC mortality is high among men, and most deaths occur in low‐ and middle‐income countries (LMICs) in Latin America, Eastern Europe, Northern Africa, and Eastern and Southeastern Asia [9, 10]. In the last two decades, LMICs and emerging economies have witnessed a growing burden of LC due to a lack of symptom awareness, symptom misappraisal, rising trends in smoking, reluctance to engage in preventive behaviors, high incidence of air pollution, and pulmonary infections. Barriers to early diagnosis of LC in LMICs include poor access to recommended care options, poor referral pathways, lack of effective LC screening programs, and significant disparities in implementing biomarker testing [5, 9, 11, 12, 13, 14, 15]. The diagnosis, staging, and treatment of LC require multiple healthcare providers with different areas of specialization. Furthermore, the evolving diagnostic and treatment landscape has added complexity to the decision‐making process. Coordination strategies through a multidisciplinary team (MDT) care approach have been identified as cornerstones in LC management [13, 14, 16, 17, 18]. Members of an LC MDT typically include a respiratory physician, medical oncologist, radiation/clinical oncologist, thoracic surgeon, pathologist, dedicated chest radiologist, nuclear medicine physician, palliative care physician, clinical pharmacist, and nurses [18, 19]. Over the years, patient‐centric, specialized, integrated MDT evaluations (e.g., clinic‐based and tumor board) have been recommended by various international guidelines (National Comprehensive Cancer Network [NCCN], National Institute for Health and Care Excellence, and European Society for Medical Oncology) to help improve the efficiency of care, optimize treatment plans, and facilitate timely referrals [13, 18, 20, 21, 22, 23, 24, 25]. Figure 1 lists key elements of the MDT approach to LC care and considerations of the MDT for establishing optimal care pathways [13, 14, 16, 17, 18].

MDT care, when implemented early, allows for the introduction of adequate diagnostic and clinical staging procedures, molecular tests, and specialist care [26]. MDT meetings help reduce variations in LC care, particularly for those from rural and remote areas [27]. Delivering high‐quality care to patients with LC through an MDT care approach remains challenging in LMICs. Furthermore, variations exist in LC management across various LMICs and emerging economies. Identifying, analyzing, and reporting barriers to LC care are essential steps toward improving the quality of care and implementing effective corrective interventions in existing LC care processes in these countries. In this article, we evaluated the impact of the MDT care approach in ESLC and listed guideline recommendations on the positioning of MDT care within a comprehensive approach to LC management. We explored barriers to MDT implementation that are unique to the care of patients with LC in LMICs and emerging economies and summarized expert recommendations on strengthening MDT care pathways for ESLC (NSCLC: clinical stage Ib to IIIa and small cell LC [SCLC]: limited stage) in these countries.

## 2 Methodology

A panel of 12 experts from 11 countries (namely Brazil, Chile, Malaysia, Colombia, Egypt, Mexico, Türkiye, Argentina, China, Vietnam, and India) contributed to the development of evidence‐informed recommendations. The experts were selected based on their recognized expertise in LC management, understanding of healthcare systems, and/or clinical research in the field. A narrative literature review was carried out based on data from the PubMed database (between January 2001 and May 2025) to identify existing gaps in ESLC management and barriers to MDT implementation in LMICs and emerging economies. Various combinations of keywords such as “lung cancer,” “early‐stage,” “multidisciplinary team,” “composition,” “care approach,” “diagnosis,” “treatment,” “management,” “outcomes,” “survival,” “care pathway,” “low‐ and middle‐income countries,” “emerging economies,” “challenges,” “barriers,” “recommendations,” “consensus,” and “guidelines” were used. Appropriate variations in search phrases and Boolean operators (AND, OR) were considered. Findings were organized thematically to: (i) evaluate the impact of MDT care on ESLC management; (ii) identify factors influencing the management of ESLC and MDT care implementation in LMICs and emerging economies; (iii) list priority areas for strengthening system‐wide MDT practices; and (iv) formulate a rational framework for improved standardized MDT care in ESLC (RISE‐LC), which can be implemented in these nations.

The RISE‐LC framework was developed through an iterative expert‐driven process informed by the literature review and virtual discussions. In round 1, the experts independently reviewed the findings from the literature and provided input on key system‐level gaps, challenges, and priorities for MDT care in ESLC. These inputs were organized into eight thematic domains:

- Early detection and referral systems
- MDT structure and expertise
- Health system and infrastructure
- Diagnostic and staging coordination
- Treatment planning, delivery, and patient care reevaluation
- Data systems, communication, and documentation
- Standardization of MDT processes and quality improvement
- Workforce and capacity building

Responses from round 1 were collated and assessed to identify areas of agreement and divergence. Recommendations were drafted with consideration of clinical applicability, feasibility across diverse resource settings, and alignment with real‐world multidisciplinary practice for ESLC. In round 2, the preliminary framework structure was shared with the panel for further refinement, clarification, and expansion. In round 3, the revised framework was circulated for final review, with experts providing confirmatory feedback and minor revisions to achieve convergence of opinion.

## 3 Results

### 3.1 Impact of MDT Care on ESLC Management

Several retrospective and prospective studies on the impact of MDT presentation have suggested that patients with ESLC benefit in completeness of clinical staging, timeliness of care, receipt of surgery, and prolonged survival [28, 29, 30, 31, 32, 33]. Conron et al. reported that patients with ESLC (stages I and II NSCLC; limited‐stage SCLC) in Australia managed by the multidisciplinary clinic (MDC) received timely diagnosis and staging [28]. Around 91% of patients received surgery within 6 weeks of initial MDC consultation, and none had to wait for > 8 weeks [28]. Stevens et al. studied the management of early‐stage I/II NSCLC in New Zealand and found that discussion during an MDT meeting was associated with an increased likelihood of undergoing curative treatment (*p* < 0.001) [34]. An analysis of the Department of Veterans Affairs Central Cancer Registry, US, compared utilization of radiation therapy (RT) for unresected stage I/II NSCLC, stage IIIA NSCLC, and limited‐stage SCLC based on presentation at an LC‐specific multidisciplinary tumor board (MTB; general MTB or no MTB) [35]. Discussion at a general MTB resulted in an increase in RT utilization for unresected stage I or II NSCLC [35]. Furthermore, patients were more likely to undergo chemotherapy (ChT) and RT for unresected stage IIIA NSCLC or limited‐stage SCLC if they were discussed in a general or LC‐specific MTB than if they were not discussed at an MTB [35]. Peckham et al. reported a 37% increase in the number of diagnoses (pre‐ to postimplementation) in patients with stages I and II NSCLC after implementation of an MTB and the creation of clinical pathways by the oncology nurse navigator in the US [29]. Additionally, surgical treatment increased by 5% and stereotactic body radiation therapy (SBRT) utilization by 1%. Improved adherence to NCCN guidelines and standardized care was also reported [29].

Studies report that MDT discussion reduces unnecessary procedures and improves patient management. Freeman et al. found that presentation at the multidisciplinary thoracic malignancy care conference (MCC) significantly improved adherence to NCCN guidelines for staging and treatment in patients with NSCLC (*p* < 0.0001) in the US [30]. A significantly reduced time from pathologic diagnosis to treatment (ChT, RT, or surgery) in the MCC group versus the non‐MCC group (mean: 17 vs. 29 days; *p* < 0.0001) was noted [30]. The study highlighted that a significantly higher number of patients with stage IIIA NSCLC underwent surgical resection in the MCC group (*p* = 0.0079) [30]. MCC was associated with significant cost savings (*p* < 0.0001) in patients with NSCLC (stages I–III) due to adherence to NCCN guidelines for diagnosis and staging and reduced facility costs [30]. Additionally, palliative care was frequently offered to patients in the MCC group versus the non‐MCC group [30]. Voong et al. investigated healthcare expenditure incurred in the diagnostic period before treatment initiation by patients with stage II to III NSCLC managed in a single‐day thoracic oncology MDC versus those managed in non‐MDC settings in the US [36]. The study found an average decrease of 23% in overall charges incurred per patient for those seen at the MDC versus those seen at the non‐MDC settings [36]. The average number of provider visits per patient was reduced at the MDC (4.8 vs. 6.8), which led to a 50% decrease in provider charges per patient (*p* < 0.01) compared with that in non‐MDC settings [36].

MDT‐based patient care has been shown to improve the survival of patients with ESLC. These benefits can be attributed to the greater effectiveness of case management, improved care coordination, and reduced variation in care. Saeteng et al. showed reduced overall mortality (6.5% vs. 35%; *p* < 0.001) in the MCC group with a larger proportion of patients with stage I and II resectable NSCLC compared with that in the non‐MCC group (78.6% vs. 59.69%) in Thailand [31]. The MCC group showed improved 5‐year overall survival (OS) across stages Ib through IIIa NSCLC compared with the non‐MDT group (Ib: 95% vs. 76%; IIa: 100% vs. 75%; IIb: 92% vs. 69%; IIIa: 92% vs. 62%) [31]. Shorter waiting times for investigation (bronchoscopy: 3 vs. 12 days; pathological results: 7 vs. 13 days) and surgical scheduling times (18 vs. 25 days) contributed to survival benefits in the MCC group [31]. Hung et al. reported that MDT discussion resulted in a significant survival benefit in patients with stage III NSCLC (41.2 vs. 25.7 months; *p* = 0.018) in Taiwan [32]. The median survival of patients with stage IIIA tumors was 39.4 months [32]. Valsecchi et al. highlighted that the 5‐year OS rate was higher in the post‐MDT cohort than in the pre‐MDT cohort for patients with resected (*p* = 0.004) and nonresected (*p* = 0.0001) stage III LC (predominantly stage IIIA: 73.8%) in Italy [33]. In the post‐MDT cohort, the 5‐year OS rates were 52.2% for patients treated with trimodality therapy (ChT + surgery + RT), 40.2% for those receiving systemic therapy, and 29.7% for patients who underwent surgery with or without RT [33]. However, in patients with SCLC, no substantial difference in 5‐year OS rates was observed between the pre‐MDT and post‐MDT study groups [33].

Various societies and consensus groups recommend the MDT approach Table 1 at multiple levels of the LC care continuum (i.e., screening, referral, diagnosis and staging, treatment, postsurgery rehabilitation, and palliative care) [20, 21, 22, 25, 37, 38, 39, 40, 41, 42, 43, 44, 45]. Multidisciplinary care is important in providing optimized patient‐centric care for patients with LC, particularly in complex cases requiring multimodal treatment. In resource‐limited countries, a basic/core configuration of a care framework needs to be determined based on resource availability (Table S1) and the cost of care [46]. If basic resources for cancer‐specific treatment are unavailable, palliative and supportive care should be provided.

**TABLE 1:** Guideline/consensus recommendations on the positioning of MDT care in ESLC management.

NCCN (guideline) [20, 21]	*Diagnosis*: “Decisions about the optimal diagnostic steps for suspected stage I–III NSCLC should be made by thoracic radiologists, interventional radiologists, thoracic surgeons, and pulmonologists.” “If a preoperative or intraoperative tissue diagnosis appears risky or unreliable, MDT evaluation is recommended to determine the safest and most efficient approach for biopsy or to provide a consensus that a biopsy is too risky or difficult.” “MDT evaluation is recommended before treatment for NSCLC patients with stage IIB and stage IIIA tumors who have different treatment options (surgery, RT, or ChT).” *Treatment*: “TSOC should be part of evaluating any NSCLC patient being considered for curative local treatment.” “In cases where SBRT is considered for high‐risk or borderline operable patients, MDT evaluation, including that by a radiation oncologist, is recommended.” “MDT evaluation is recommended before surgery for limited‐stage SCLC.” “Radiation oncology input, as part of an MDT evaluation or discussion, should be provided for all SCLC patients early in the determination of the treatment strategy.”
ASCO (guideline) [37, 38]	“Patients with stage I NSCLC should be evaluated by a thoracic surgeon, preferably within MDT to determine operability.” “For patients with high operative risk stage I NSCLC, discussions about SBRT as a potential alternative to surgery are encouraged within the MDT.” “For patients who have suspected or confirmed stage III NSCLC, MDT discussion should occur before initiating any treatment plan.”
ESMO (guideline) [25]	*Diagnosis*: “Pretreatment pathological diagnosis is strongly recommended for all patients before SBRT unless an MTB considers that the RBR of the procedure is unacceptable.” *Treatment*: “Adjuvant ChT should be offered to patients with resected stage II and III NSCLC and can be considered in patients with resected stage IB disease and a primary tumor sized > 4 cm. Preexisting comorbidity, time from surgery, and postoperative recovery need to be discussed by an MTB.”
NICE (guideline) [22]	*Diagnosis*: “Care of all people with a working diagnosis of LC should be discussed at an LC MDT meeting.” *Treatment*: “MDT teams that provide ChRT with surgery should have expertise in combined therapy and all the individual components.” *Supportive and palliative care*: “Weight loss, loss of appetite, depression, and difficulty swallowing should be managed by MDT, which includes supportive and palliative care specialists.”
UK Lung Cancer Coalition [39]	*Referral criteria*: “Reports of all chest X‐rays and CT scans where the possibility of a diagnosis of LC is raised should be sent urgently both to the referring clinician and the LC team. The interval between GP referral and first attendance in the specialist clinic should be as short as possible (not more than a week).”
Management of Early‐Stage Resected NSCLC, Saudi Arabia (Consensus) [40]	*Diagnosis*: “The MDT approach is recommended for the workup and staging according to the availability and expertise in early‐stage resected NSCLC (Ib–IIIa^a^).” *Treatment*: “MDT should decide about adjuvant ChT considering preexisting comorbidities, time from surgery, and postoperative recovery.”
ATORG (Consensus) [41]	*Treatment*: “All stage III NSCLC patients considered potentially resectable should be discussed with the MDT. If surgery is considered, it should be preplanned with either neoadjuvant ChT, ChRT, or TKI and should ideally be performed in high‐volume centers with experienced trimodality teams.” “Complex cases are to be discussed at MDT discussions for consensus on combined‐modality approaches.”
Lung Cancer Screening in Asia (Consensus) [42]	“The involvement of MDT in LC screening and management of incidental pulmonary nodules is recommended.”
NCCN Harmonized Guidelines for SSA (Guideline) [43, 44]	“Lower‐level care options should be considered only when referral or access to higher levels is not possible. Delays in treatment reduce the effectiveness of treatment. MDT care is always recommended.”
Management of Nonmetastatic NSCLC in MEA (Consensus) [45]	“Decisions about the optimal diagnostic steps for suspected stage I to III NSCLC should be made by thoracic radiologists, interventional radiologists, thoracic surgeons, and pulmonologists.” “Surgeons, medical oncologists, and molecular biologists should discuss the individual case to decide on an appropriate approach for surgical management and adjuvant therapy.”

Abbreviations: ASCO, American Society of Clinical Oncology; ATORG, Asian Thoracic Oncology Research Group; ChRT, chemoradiotherapy; ChT, chemotherapy; CT, computed tomography; ESLC, early‐stage lung cancer; ESMO, European Society for Medical Oncology; GP, general practitioner; LC, lung cancer; MDT, multidisciplinary team; MEA, Middle East and Africa; MTB, multidisciplinary tumor board; NCCN, National Comprehensive Cancer Network; NICE, National Institute for Health and Care Excellence; NSCLC, non‐small cell lung cancer; RBR, risk–benefit ratio; RT, radiation therapy; SBRT, stereotactic body radiation therapy; SCLC, small cell lung cancer; SSA, Sub‐Saharan Africa; TKI, tyrosine kinase inhibitor; TSOC, thoracic surgical oncology consultation.

^a^ Stage IIIa includes T1a to c N2 M0, T2a to b N2 M0, T3 N1 M0, T4 N0 M0, and T4 N1 M0.

### 3.2 Factors Influencing the Management of ESLC and MDT Care Implementation in LMICs and Emerging Economies

Studies from Nigeria, India, and KwaZulu‐Natal, South Africa, mention that low health literacy, misconceptions about LC origins, cultural beliefs, and a lack of public education campaigns contribute to poor cancer awareness and diagnostic delays [47, 48, 49, 50, 51, 52, 53, 54]. Most patients with LC in LMICs and emerging economies (e.g., India, Brazil, Argentina, Colombia, Malaysia, Korea, and China) present with advanced disease (clinical stage IIIB–IV) at the time of diagnosis [42, 55, 56, 57, 58, 59, 60, 61, 62, 63]. Bhosai et al. reported that sociodemographic factors and beliefs about cancer can cause delays in seeking medical attention [64]. In several LMICs, health systems lack adequate infrastructure (diagnosis, treatment, and palliative care) and financial resources for equitable patient‐centric cancer care. Studies from Africa (e.g., Ethiopia and Uganda), Egypt, the Philippines, Syria, Jordan, and Colombia highlight that weak organizational structure, fragmentation of the financing and health system, oncology workforce deficits, unawareness of the potential impact of MDTs, and lack of coordination between the different specialties are some of the barriers to implementation of MDT cancer care in LMICs [12, 65, 66, 67, 68, 69, 70, 71, 72, 73, 74]. For example, Ramesh et al. reported a significant oncology workforce deficit in India, with an oncologist‐to‐patient ratio of approximately 1:2000, each oncologist managing around 475 new patients annually [75]. In addition, nearly 95% of referral cancer centers are concentrated in urban areas, resulting in extended waiting times and limited access to specialist cancer care for patients in rural and semi‐urban regions [75]. Likewise, Murillo et al. reported that Colombia has a limited radiation oncology workforce and infrastructure, with 101 radiation oncologists, 53 RT services, and 28 brachytherapy centers [72]. Based on an estimated 66 000 cancer cases requiring RT in 2018 and a staffing benchmark of 1 specialist per 250 new cancer cases, the projected requirement of approximately 265 radiation oncologists highlights a substantial workforce deficit in RT services [72]. Furthermore, disparities in access to specialty consultations, lack of integrated systems to access medical records, and lack of national or regional MDT guidelines (e.g., criteria for patient inclusion, referral, collection of treatment/diagnostic history, and MDT quality control and assessment) further hinder optimal cancer care [76]. Clinical trials and high‐quality research are limited in LMICs due to several challenges, such as a lack of research culture, infrastructure, expertise, and funding [77, 78]. Certain LMICs do not have adequate cancer registries (e.g., Pakistan and Libya) to identify the processes and outcomes of cancer management [79]. Poor awareness of physicians' medicolegal responsibilities and inadequate secure information infrastructure for data security (patient confidentiality, privacy) remain roadblocks to patient‐centric multidisciplinary care in LMICs [80, 81].

Access to thoracic imaging (notably computed tomography [CT] and positron emission tomography [PET]) is crucial for pulmonologists and thoracic surgeons, especially within the MDTs. While this access is generally easier in high‐income countries, LMICs often face significant challenges in securing these resources. Early LC care in LMICs (e.g., Egypt, Pakistan, and India) is hindered due to the inaccessibility of diagnostic investigations, inadequate utilization despite awareness, physician unawareness/lack of screening guidelines, lack of insurance coverage, and delayed referral for further testing [82, 83, 84, 85, 86, 87]. Yurdakul et al. reported frequent diagnostic and treatment delays in patients with early‐stage NSCLC in Turkey [88]. Furthermore, studies highlight that LC may be misdiagnosed as pulmonary tuberculosis (TB), particularly in regions where TB is prevalent (e.g., Brazil, Peru, India, Bolivia, Ecuador, and Vietnam), due to clinical and radiological similarities leading to a delay in diagnosis [57, 89, 90, 91, 92, 93, 94]. Studies from India report that insufficient knowledge of LC among physicians, misinterpretation of chest radiographs, initiation of empirical anti‐TB treatment for suspicious opacities on chest radiographs without proper evaluation (notably CT scan, sputum cytology, and bronchoscopy), and the coexistence of pulmonary TB and LC led to substantial delays in LC treatment initiation [60, 92, 95, 96]. A pulmonologist in the MDT can help navigate the complexities and offer expertise in cases where both LC and TB are detected simultaneously, helping to determine the optimal management strategy and minimize potential treatment delays.

Routine biomarker testing for LC (programmed cell death protein 1 [PD‐1]/programmed death‐ligand 1 [PD‐L1], epidermal growth factor receptor [*EGFR*], Ki‐ras2 Kirsten rat sarcoma viral oncogene homolog [*KRAS*], v‐raf murine sarcoma viral oncogene homolog B1 [*BRAF*], anaplastic lymphoma kinase [*ALK*], and ROS proto‐oncogene 1 receptor tyrosine kinase [*ROS1*]) in biopsied tissue/liquid biopsy has been suboptimal in LMICs [15]. Clinical adoption of molecular testing is heterogeneous in various LMICs, largely due to diversity in access to molecular testing and approved therapies (e.g., Egypt, Indonesia, Myanmar, and Serbia), limited health insurance coverage, and inadequate financial assistance programs [15]. In some LMICs, pharmaceutical companies sponsor certain molecular testing (e.g., Serbia and Egypt) during clinical trials or routine clinical testing; however, large mutation panels are usually outsourced and mostly covered by patients [15].

Curative treatment for LC, including surgery, RT, and systemic therapy, involves a multidisciplinary approach and coordinated care. Experienced oncologists or thoracic surgeons (e.g., Taiwan and India) and access to palliative care centers and postsurgery rehabilitation services are limited (e.g., isolated, locoregional services in Brazil, Kuwait, Lebanon, and Mexico) in some LMICs and emerging economies [54, 97, 98]. Furthermore, the availability of RT is limited in LMICs (e.g., India, Ghana, Taiwan, and Indonesia), and patients face long wait periods [99]. Cruz‐Lim et al. reported that reimbursement issues, lack of training, and inadequate treatment equipment resulted in the underutilization of SBRT and radiosurgery in the Philippines [100]. Due to resource constraints, chemoradiotherapy may be challenging in some settings. Multiorganizational collaboration across a diverse network of local LC care professionals and global partners, sustainable investment strategies, and locally adapted solutions can play an important role in strengthening the MDT care approach in these cases.

Table 2 consolidates the system‐level barriers into structured thematic domains to highlight the key priority areas for strengthening MDT care in ESLC. It organizes the multifactorial challenges spanning patient‐level delays, diagnostic and infrastructural limitations, workforce constraints, and gaps in coordination into actionable categories.

**TABLE 2:** Thematic domains and priority areas for strengthening MDT care in ESLC.

Early detection and referral systems	Integration of screening programs within health systems Presentation of patients at the diagnosis stage Awareness of ESLC Structure of referral pathways Coordination between primary and specialist care services
MDT structure and expertise	Access to MDT Composition of MDTs in lung cancer care Inclusion of core specialties Variation in MDT organization and functioning
Health system and infrastructure	Availability of diagnostic infrastructure Access to molecular and biomarker testing Availability of treatment infrastructure
Diagnostic and staging coordination	Organization of diagnostic workflows Staging workup Use of imaging modalities
Treatment planning, delivery, and patient care reevaluation	Selection of treatment modalities Availability of advanced treatment options
Data systems, communication, and documentation	Data sharing mechanisms across specialties and security Documentation of MDT decisions Clinical information exchange system
Standardization of MDT processes and quality improvement	Availability of MDT guidelines and protocols Standardization of clinical decision‐making processes
Workforce and capacity building	Availability of trained oncology specialists MDT training exposure and capacity building

Abbreviations: ESLC, early‐stage lung cancer; MDT, multidisciplinary team.

### 3.3 Recommendations to Strengthen MDT Care for ESLC in LMICs and Emerging Economies: Call for Action and Development of Framework

#### 3.3.1 System‐Level Recommendations

**FIGURE 2:**
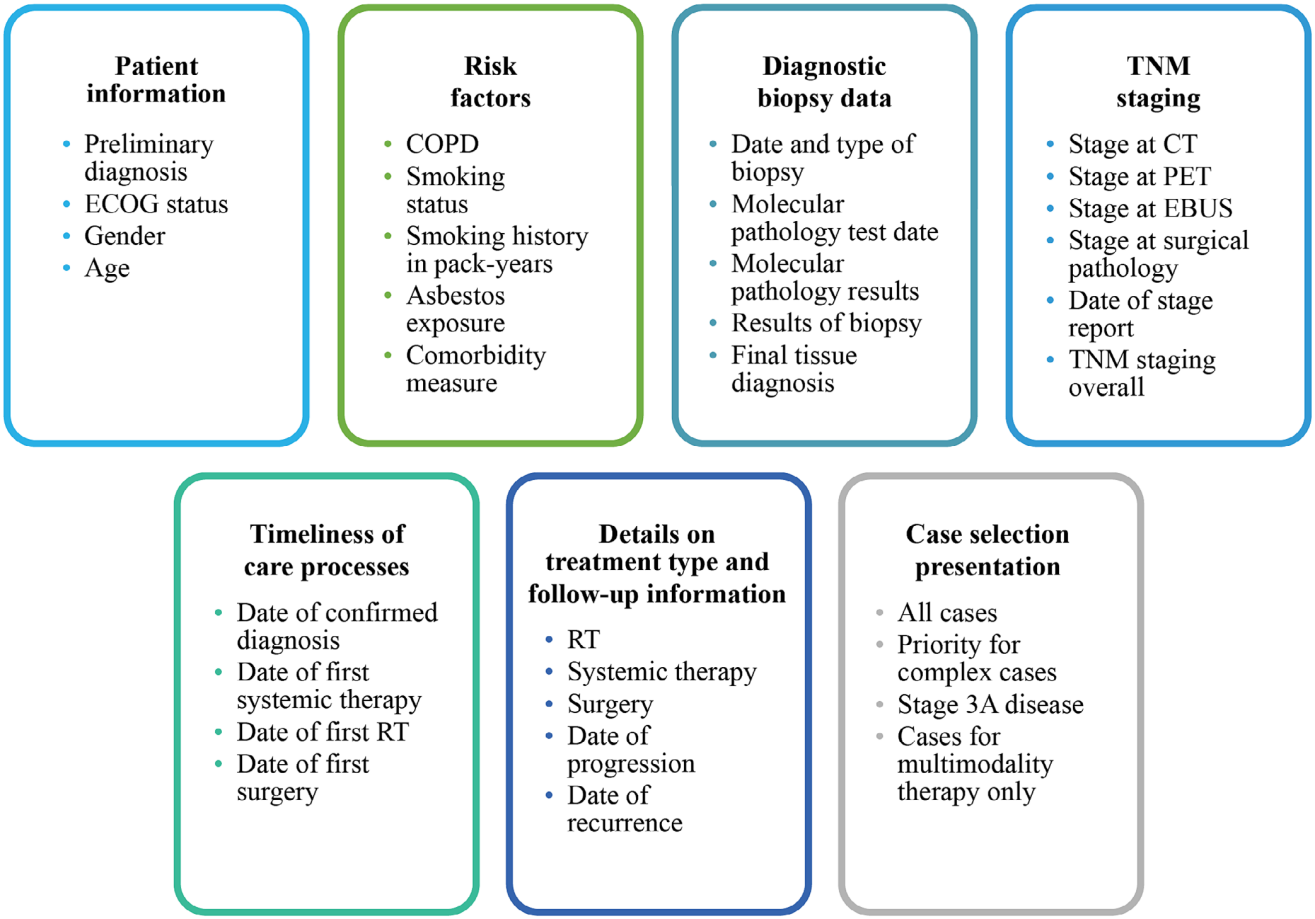
Data set for standardization of MDT data collection and internal MDT review. ChT, chemotherapy; COPD, chronic obstructive pulmonary disease; CT, computed tomography; EBUS, endobronchial ultrasound; ECOG, Eastern Cooperative Oncology Group; MDT, multidisciplinary team; PET, positron emission tomography; RT, radiation therapy; TNM, tumor, node, and metastasis. Information sourced from: Stone et al. [105].

- The MDT panel for initial workup, staging, and molecular testing for early‐stage NSCLC should include thoracic radiologists, interventional radiologists, thoracic surgeons, pulmonologists, and molecular biologists before treatment is initiated [43, 45, 101]. The determination of the appropriateness of RT should be made by radiation oncologists [43, 45, 101].
- A team of thoracic surgeons, medical oncologists, and molecular biologists should be constituted for the determination of resectability, surgical staging, and pulmonary resection in patients with NSCLC [43, 45, 101]. Oncology nurse navigator support, respiratory therapists, and palliative care physicians can be valuable additions to an early LC MDT [29, 102, 103, 104]. For patients with limited‐stage SCLC, MDT evaluation is recommended before surgery [44]. To achieve effective MDT working, it is important to establish a core team that will lead and take responsibility. A core team encompassing specialists from medical oncology, nuclear medicine, radiotherapy, and surgical oncology units can be constituted for MDT meetings.
- Strengthening of health systems (i.e., infrastructure and resource availability) requires special attention in LMICs and emerging economies. This can be achieved by promoting collaboration with academic medical centers, professional societies, nongovernmental organizations, governmental organizations, and global research centers. Multiorganizational collaboration facilitates the exchange of best practices and can play an important role in strengthening local capacity. Primary care physicians should be trained to recognize symptoms, understand risk factors, and know when to refer patients with LC for screening and diagnostic testing. Collaboration and targeted investments to expand access to early screening (low‐dose CT without contrast) and diagnostic modalities integrated into primary care settings can help mitigate treatment delays in patients with ESLC. Primary care physicians can also play an important role in the early diagnosis and referral of patients with LC. Targeted investments should be directed toward developing facilities for CT, PET‐CT, minimally invasive mediastinal staging, magnetic resonance imaging (or CT of the brain), SBRT, and video‐assisted thoracic surgery lobectomy. Mediastinal staging is crucial in LC staging. Although surgical mediastinoscopy has largely been replaced by endobronchial ultrasound (EBUS)‐guided transbronchial needle aspiration (EBUS‐TBNA), access to these technologies remains limited in several LMICs; therefore, at least cervical mediastinoscopy performed by a trained surgeon must be available as a cost‐effective procedure. Biomarker testing (genetic alterations in *EGFR*, *ALK*, and PD‐L1 expression) can be done for early‐stage nonsquamous NSCLC [45, 100, 105]. For analysis, standardized facilities for immunohistochemistry, fluorescent in situ hybridization, and multiplex reverse transcription‐polymerase chain reaction should be developed [15]. Furthermore, studies have highlighted considerable variability in the adoption, implementation, and auditing of MDT care [18, 106]. In some cases, adherence to guidelines may not be consistent throughout MDTs. To address these gaps, there is a need for development of regional or national MDT guidelines that clearly define (i) patient referral criteria, (ii) core MDT composition, (iii) information‐sharing protocol for electronic medical records, (iv) data confidentiality and management, (v) quality assessment processes, (vi) patient discussion and follow‐up, pathways, and (vii) clinical audit mechanisms.
- Geographic disparities restrict access to the best specialists, particularly in LMICs and emerging economies. Virtual MDT meetings facilitated by digital technologies enable the participation of specialists from remote locations, enhancing collaboration and accessibility [77, 107]. While virtual MDT models can support improved access to expertise, strengthening structured face‐to‐face MDT meetings remains important, with hybrid models offering a pragmatic approach to ensuring both accessibility and depth of multidisciplinary decision‐making in LMICs and emerging economies [108].
- MDTs should develop information databases that record patient characteristics and risk factors, including smoking, treatments, complications, and patient‐reported outcomes. Access to streamlined data collection strategies, standardized datasets, and integrated real‐time platforms (electronic health records, medical images, remote consultations, and monitoring) can improve the MDT decision‐making process [109, 110]. Figure 2 lists the data set for LC MDT team evaluation, considering key metrics (LC stage, risk factors, wait time, and treatment type) [109]. Policy barriers obstructing data sharing must be dismantled, fostering an environment conducive to secure data exchange among stakeholders. Efficient MDT operation can be ensured by the inclusion of imaging, pathology, and biopsy standards in guidelines, considering best practices and local and regional expertise. It is essential to implement a transparent and ethical data sharing practice among relevant stakeholders. Data on care processes, notably time to diagnosis, time to first treatment, and time to relapse, should be documented and reviewed periodically in MDT discussions [109].
- Educating professionals about the benefits of MDC and the provision of appropriate remuneration plans can improve the implementation of multidisciplinary care [111, 112].
- Regular audits can be conducted to confirm that treatment recommendations match current best practices [113, 114].
- When MDTs are unable to discuss every single case due to logistical constraints, it is crucial to focus on complex cases while considering current standards, resource availability, the latest clinical research, and evidence [18].

#### 3.3.2 Clinician‐Level Recommendations

- Financial disincentives, lack of motivation, and time constraints are barriers to full participation in MDT meetings [111]. Establishing strong leadership and promoting a culture of collegiality and trust enables MDT members to participate actively in MDT discussions [112].
- Healthcare professionals should allocate specific time for interdisciplinary meetings and ensure all necessary information is available for effective case discussion at MDT meetings [112].

**FIGURE 3:**
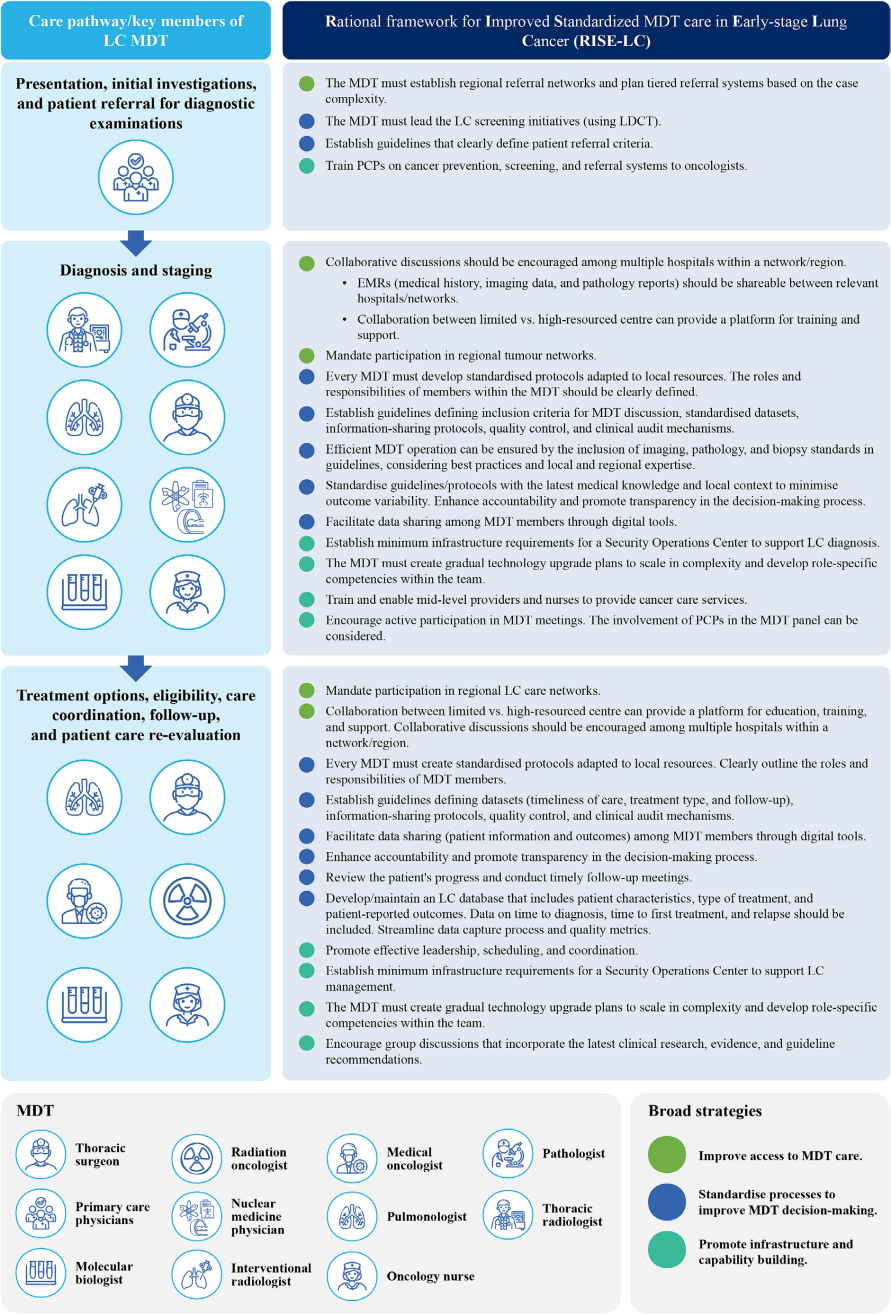
Key strategies for strengthening MDT care for ESLC in LMICs and emerging economies. EMR, electronic medical record; LC, lung cancer; LDCT, low‐dose computed tomography; LMICs, low‐ and middle‐income countries; MDT, multidisciplinary team; PCP, primary care physician.

Figure 3 depicts a **R**ational framework for **I**mproved **S**tandardized MDT care in **E**S**LC** (**RISE‐LC**) that can be adopted in LMICs and emerging economies. This framework focuses on (i) establishing robust referral systems, (ii) promoting collaboration among multiple hospitals within a network/region, (iii) fostering participation in regional tumor networks that provide specialized cancer care/research, (iv) setting role‐specific competencies within the MDT, (v) establishing guidelines defining inclusion criteria for MDT discussion, (v) use of standardized datasets and information‐sharing protocols, (vi) implementing quality control measures, (vii) providing comprehensive training that leads to continual improvement and advancement in the field, and (viii) establishing clinical audit mechanisms for effective LC care.

## 4 Conclusion

Delivering high‐quality cancer care to patients with LC through an MDT care approach remains challenging in LMICs and emerging economies. Weak organizational structure, oncology workforce deficits, unawareness of the potential impact of MDT, disparities in access to specialty consultations, lack of coordination, and inadequate secure information infrastructure for data security hinder optimal cancer care. Integration of primary care delivery platforms with specialized oncology care will be critical to curb the rising tide of LC in resource‐limited settings. There is a need to formulate resource‐stratified national/regional MDT guidelines for streamlining management and steering treatment‐related decisions, considering local constraints, resources, and infrastructure and providing a training and collaborative research platform. Further research into economic evaluations of MDT care in LMICs could reassure policymakers that this model of care can ensure optimal utilization of resources and better use of funds across health systems in LMICs and emerging economies for ESLC. Lastly, establishing institutional‐level LC registries capturing clinical profiles, treatments, and patient‐reported outcomes at the level of individual hospitals or cancer centers represents a practical and actionable first step toward improving MDT data systems in resource‐constrained settings. Such registries can provide immediate insights to local MDTs for quality improvement and can serve as building blocks for future regional or national data infrastructure.

## Author Contributions

**Luiz Henrique Araujo:** conceptualization, methodology, supervision, visualization, writing – original draft, writing – review and editing. **Francisco J. Orlandi:** funding acquisition, visualization, writing – original draft, writing – review and editing. **Trung Lam Quoc:** writing – original draft, writing – review and editing. **Anusheel Munshi:** writing – original draft, writing – review and editing. **Yasser Elkerm:** writing – original draft, writing – review and editing. **Jorge Alatorre‐Alexander:** writing – original draft, writing – review and editing. **Xiao Li:** writing – original draft, writing – review and editing. **Lucia Viola:** writing – original draft, writing – review and editing. **Sevin Başer Öncel:** writing – original draft, writing – review and editing. **Adli Azam:** writing – review and editing, writing – original draft. **Gustavo A. Lyons:** writing – original draft, writing – review and editing. **Francisco Gonzalez:** writing – original draft, writing – review and editing.

## Funding

This study was funded by AstraZeneca in accordance with Good Publication Practice (GPP) guidelines 2022. The funding source had no role in contribution toward study design; writing of the manuscript, in the collection, analysis, and interpretation of data; and in the decision to submit the paper for publication.

## Ethics Statement

The authors have nothing to report.

## Consent

The authors have nothing to report.

## Conflicts of Interest

Araujo Luiz Henrique received consulting fees from MSD, Roche, AstraZeneca, BMS, and Lilly, and payment/honoraria for lectures from MSD, Bristol Myers Squibb AstraZeneca, Boehringer, Merck, Roche, Pfizer, and Lilly. Francisco J. Orlandi received institutional grants/contracts from AbbVie, Amgen, Astellas Pharma, AstraZeneca, BMS, Daiichi Sankyo, Gilead, MSD, Novartis, Pharma Mar, Regeneron, Roche, and Sanofi; consulting and speaker fees/honoraria from AstraZeneca, BMS, Roche, and MSD; and support for attending meetings/travel from Roche and AstraZeneca. Adli Razi discloses consulting fees as a cardiothoracic surgeon, honoraria for lectures/educational events from Orion Pharma, and Dr. F. Kohler Chemie Pharma; and participated in an advisory board for AstraZeneca. Lucia Viola received consulting and speaker fees, meeting/travel support, and advisory board participation from AstraZeneca and MSD. Yasser Elkerm received speaker payment/honoraria from AstraZeneca, Roche, Eva Pharma, Merck, and Astellas Pharma. Jorge Alatorre‐Alexander received consulting fees, speaker payment/honoraria, support for meetings/travel, and participation on advisory boards from AstraZeneca, Roche, Boehringer Ingelheim, MSD, BMS, Takeda, Pfizer, Janssen, Amgen, and Adium. Sevin Baser Oncel has no conflicts of interest to disclose. Gustavo A. Lyons received speaker payment/honoraria from Roche, Pfizer, and AstraZeneca. Xiao Li has no conflicts of interest to disclose. Trung Lam Quoc received consulting fees, lecture honoraria, meeting/travel support, and advisory board participation from AstraZeneca. Anusheel Munshi has no conflicts of interest to disclose. Francisco Gonzalez is an AstraZeneca employee.

## Supporting information


**Table S1:** Resource stratification.

## Data Availability

Data sharing not applicable to this article as no datasets were generated or analysed during the current study.
