# Supplementary material for: Multidisciplinary Team Care for Early‐Stage Lung Cancer in Low‐ and Middle‐Income Countries and Emerging Economies: Current Position and Call for Action
Source: Thorac Cancer. 2026 Aug 18;17(16):e70372. doi: 10.1111/1759-7714.70372 (PMC13483319; doi:10.1111/1759-7714.70372)
Supplement: Supplementary file 1 [file TCA-17-e70372-s001.doc]

**Supplementary Table S1:** Resource stratification

| **“Basic”** | “Include essential services to provide a basic minimal SOC that improves disease-specific outcomes”. |
| --- | --- |
| **“Core”** | “Include services provided in the basic resources framework plus additional services that provide major improvements in disease outcomes (survival) and that are not cost-prohibitive”. |
| **“Enhanced”** | “Include services provided in the core resources framework plus additional services that provide lesser improvements in disease outcomes and/or services that provide major improvements in disease outcomes but are cost-prohibitive in lower-resource settings”. |

*SOC: Standard of care.*
